# 

**Published:** 1867-07

**Authors:** 


					﻿WANTED.
The February and March numbers of the current volume of
the Dental Register have become exhausted, and we are
anxious to obtain a few copies. Any persons having these
numbers who will spare them, will please forward them to us
by mail immediately, and we will either pay for them, or
return any other number, as the parties may desire.
J. Taft, Publisher.
The Dental Register
OF THE WEST,
Issued Monthly, at $3 a Year in Advance.
DEVOTED TO THE INTERESTS OF TIIE DENTAL PROFESSION.
EDITED BY J. TAFT AND G. WATT.
The volume commences in January aud closes in December.
The Register being issued monthly is always fresh, therefore indispensable to the practi-
tioner who desires to keep posted up with the new inventions and improvements which are
daily developed. Neither expense nor effort will be spared to give value and variety to its
contents. Articles will be illustrated with well executed engravings whenever they will add
to the interest or assist in explanation.
Its extensive circulation and frequency of issue makes it one of the BEST DENTAL
ADVERTISING MEDIUMS in the United States.	fc
RATES OF ADVERTISING.
One page, 1 year, $50. Half page, 1 year, $30. Quarter page, or card, 1 year $20.
All advertising bills payable in advance, or at furthest after the issue of the first number
containing the advertisement. AU transient advertisements to be paid for in advance.
fi®- CONTRIBUTIONS SOLICITED.
Address, J. TAFT, or “DENTAL REGISTER," Cincinnati, Ohio.
Busiuess'Communications to
-V- TAFT, f u.blish.er',
No. 238 Fourth St., between Plum and Central- Avenue-.
				

